# Editorial: Healthy organizations and social capital: promotion of wellbeing

**DOI:** 10.3389/fpsyg.2023.1204837

**Published:** 2023-06-15

**Authors:** Marta Gil-Lacruz, Gregorio Gimenez, Isabel Saz-Gil, Ana I. Gil-Lacruz

**Affiliations:** ^1^Department of Psychology and Sociology, Universidad de Zaragoza, Zaragoza, Spain; ^2^Department of Applied Economics, Universidad de Zaragoza, Zaragoza, Spain; ^3^Department of Business Organisation and Management, Universidad de Zaragoza, Zaragoza, Spain

**Keywords:** healthy organizations, social capital, corporate social responsibility, wellbeing, labor health

On paper, we all recognize that corporate social responsibility (CSR) promotes sustainable development, increases the satisfaction and loyalty of stakeholders and, consequently, improves the value of the organization. However, who has not thought that CSR is simply a corporate effort to improve the public image of the company? Often, CSR programs are decoupled from the core business and limited to token and marketing gestures. The dispersion in its use and in the definition of who its benefactors are means that it runs the risk of becoming a new trend without content.

Today more than ever, VUCA environments (acronym used to describe the Volatility, Uncertainty, Complexity and Ambiguity that occurs in the markets), impose on companies the need to adopt strategies that, in addition to providing them with a competitive advantage over other organizations promote its sustainability. It is essential that companies are able to retain talent and maintain the high motivation of their workers. Labor commitment facilitates the involvement of employees, both with their work and with the objectives and values of the entity in which they are integrated. This is where CSR can play a prominent role, because with proper CSR management by business leaders, the labor commitment of workers can be improved (López-Concepción et al., 2021).

Creativity, motivation and the desire to progress in the workplace require a good state of health, but not only that. More than 1 million people do not go to work on average every day. Among them, 74% were absent due to temporary disability, while the remaining 26% did so despite not being on sick leave (Randstat, 2022).

The definitions of corporate social responsibility (CSR) and Strategic Management of Human Resources (SMHR) continue to be subject to a wide and varied set of challenges (green management, sustainability, commitment, performance, satisfaction, etc.) and to multiple interpretations, as many or more, as types of agents intervene (Herrera and de las Heras-Rosas, 2020). Therefore, the potential development and measurement of the effect and consequences of these interventions have not been sufficiently explored (Herrera and de las Heras-Rosas, 2020; López-Concepción et al., 2021).

In 2018, the Global Reporting Initiative (GRI), an independent international organization that helps companies and other entities take responsibility for their impacts by providing them with a common global language to communicate them, defined a standard for companies to share, in a coordinated manner, their initiatives in relation to the promotion of workers' health. These initiatives go beyond the “traditional” risk prevention requirements for safety and health at work. They cover things like smoking cessation programs or free workplace health screenings. Thanks to these initiatives, private actors are adopting voluntary standards for their companies to intervene in areas of public health that were traditionally associated with public decision makers (Global Reporting Initiative, 2018). The Global Reporting Initiative (2018), although it constitutes an important advance in the matter, also illustrates the lack of coordination among the agents involved. Under this background, the path toward “corporate responsibility in health” promises to be long and arduous (Brassart-Olsen, 2020). Among other issues, a consensus on a common evaluation model and quality standards is the first step. In this sense, the European Commission proposes as a progress strategy, the design and evaluation of a set of Key Performance Indicators (European Commission, 2020).

This long-term horizon contrasts with the urgency of giving a coordinated response, which transcends national borders, to the needs imposed by the global health crisis of COVID-19 (Gorgenyi-Hegyes et al., 2021). To help companies make strategic decisions, academics, business leaders, and government legislators need to assess the effectiveness of CSR and SMHR policies (Mahmud et al., 2021).

The complexity of the issue and its multiple aspects require further research on the consequences of implementing adequate health strategies at workplace, as well as those derived from their absence or from incorrect corporate policies. This issue on healthy organizations and social capital includes 10 research articles that advance the state of the art in this direction. Reading these articles will allow us to understand how working conditions, and in special social support, feedback, task significance, task Identity, and autonomy impact positively the probability of being in the happy–productive pattern. At workplace, social support practices and sustainable leadership foster undoubtedly workers' wellbeing. It is confirmed that burnout has a negative impact on personal and organizational goals, but that this impact could be moderated with an adequate promotion-focused job crafting. Among causes of distress, high stress and poor sleep quality require special attention, and specific interventions need to be implemented. Among other actions, mindfulness-based interventions may positively impact employees' and managers' mental health skills and social relations at work. Aspects such as the relevance of diversity in positions of highest responsibility or which human values reinforce workers' involvement in CSR are other topics that are also addressed in this issue. Finally, a systematic review of 27 articles concludes that CSR and work health promotion have beneficial reciprocal effects.

## Author contributions

All authors listed have made a substantial, direct, and intellectual contribution to the work and approved it for publication.
